# Editorial: The war in Ukraine: impact on mental health on a global level

**DOI:** 10.3389/fpsyg.2023.1226184

**Published:** 2023-07-25

**Authors:** Mona Vintilă, Argyroula Kalaitzaki, Maria Nicoleta Turliuc, Cosmin Goian, Otilia Ioana Tudorel

**Affiliations:** ^1^Psychology Department, West University of Timişoara, Timişoara, Romania; ^2^Social Work Department, Hellenic Mediterranean University, Heraklion, Greece; ^3^Psychology Department, Alexandru Ioan Cuza University, Iaşi, Romania; ^4^Social Work Department, West University of Timişoara, Timişoara, Romania

**Keywords:** war, Ukraine, mental health, global level, repercussion

Since the start of the war in Ukraine on 24 February 2022, over 8.2 million Ukrainians have become refugees across Europe (Statista Research Department, 2023), in what the WHO (2023) has described as “the largest movement of people in the European Region since the Second World War”. One-third of the population has been displaced within Ukraine as internal refugees.

Due to the media coverage, the war rapidly sparked global attention and it affected people all over the world. Needless to say, that this war started while people were still stressed and exhausted from the unprecedented effects of COVID-19 and were struggling to return to a less isolated lifestyle.

As a historical background, Ukraine and Russia share over 1,000 years of history, with Ukraine being integrated into the Soviet Union in 1922 and achieving independence in 1991 (Conant, 2023).

Previous war-related literature has shown that war has multiple negative psychological consequences on people regardless of age, gender, or degree of involvement (Winter et al., 2015). PTSD occurs in one-third to one-half of adult refugees, and separation anxiety in up to 70% of refugee children (Javanbakht et al., 2021). Distress, anxiety, and depression are also common large-scale problems (Xu et al., 2023). The impact of social media and possible disinformation may add to the already existing distress (Rocha et al., 2021). Scant attempts have shown that the current war has already caused many mental health problems (e.g., Osiichuk and Shepotylo, 2020; Singh et al., 2021; Kurapov et al., 2022).

This Research Topic intended to present how this war has affected mental health and wellbeing globally and describes targeted interventions and preventative measures to help those affected. The contributions demonstrated the breadth of interest in this topic. Nine articles, by 46 authors from 12 countries and from two continents were published. The papers focused on the mental health of Ukrainians who remained in their home country, internal and external refugees, different age groups (e.g., children, youth, and adults) and people in different particular circumstances (e.g., pregnant women). Studies referred both to the general population, Ukrainians and civilians in other countries and people, professionals or not, involved in medical aid/psychological/social support.

Both the studies conducted by Anjum et al. and Kurapov et al. explored the mental health impact of the war in Ukrainians. The study by Kurapov et al. revealed a difference in terms of anxiety and depression between those directly exposed to the war and those indirectly exposed; gender and age were found to play a role. Those who stayed in Ukraine had significantly lower levels of anxiety, depression, stress, and trauma-related symptoms than those who moved abroad. Anjum et al. also examined refugees/asylum seekers and provided resources for those involved in helping refugees. They focused on the mental health and wellbeing of these groups, and provided strategies, action plans, and resources for those, professionals or not (e.g., psychologists, counselors, volunteers, and relief workers), involved in helping refugees.

Maftei et al. investigated the psychological outcomes of Romanian adolescents' involvement in helping Ukrainian refugees. Their main findings suggested that participants involved in helping Ukrainian refugees present higher peri-traumatic dissociative experiences and anxiety symptoms than those who are not actively involved in the helping process.

A number of papers focused on refugees, either examining their mental health or investigating interventions to support them.

Pregnant and postpartum refugee women's mental health was investigated by Rodríguez-Muñoz et al. Protective factors such as personality traits, social support, socio-demographic characteristics, and access to medical/mental health services were assessed. The findings of this study will help policymakers to develop targeted mental health prevention strategies and interventions for these particular categories of refugees.

A school-based brief psychological screening procedure of Ukrainian children and adolescents refugees in Germany was assessed by Catani et al., given that refugee minors are particularly vulnerable to mental health issues. Key elements in preventing and efficient treatment are: early detection of psychological problems, timely referrals and treatment. The results showed a good reaction to the screening process, but at the same time a considerable volume of mental health problems and high levels of distress was found.

The study by Paoletti et al. is about the impact of a neuro-psycho-pedagogical training program on the wellbeing of Ukrainian refugees. The Envisioning the Future (EF) training led to an improvement in coping strategies and increased sense of security, quality of sleep, and more frequent positive thoughts.

Costanza et al. described a meaning-centered therapy for supporting refugees. The positive results are encouraging and led the authors to conclude that this approach could offer a generalized psycho-therapeutic perspective, allowing refugees to find subjective and effective meaning in this critical situation of war.

How the war has impacted the mental health of people in other countries was also presented. Vintilă et al. examined the impact that social media consumption has on the distribution of fake news among Romanians, namely the relationship between information overload and the tendency to spread false information and the relationship between time spent online and the tendency to spread false information. Fear of war and coping strategies highly differ between those who worked with refugees and those who did not.

The mental health of Italian people not directly involved in the war were examined by Mottola et al., who analyzed the moderating roles of both risk (COVID-19) and protective (post-traumatic growth) factors on the relationship between concern about the war and levels of stress and anxiety/depression.

## Conclusion

This collection of papers makes a unique contribution to the scarce literature on the war in Ukraine, advancing our understanding of the wide-ranging effects of the war, which have extended far beyond the conflict's epicenter. The focus on the psychological impact on all the diverse groups which were investigated, on screening techniques, on the assessment of protective factors, the analysis of the efficacy of various prevention and intervention measures are the strong point of this collection of papers.

Our hope is that this Research Topic will provide useful guidelines for clinicians and public policies, as it offers preventive and intervention strategies, action plans, training programs and therapeutic alternatives. This could improve globally the refugee management and integration from a psychological point of view, improving the mental health of all those involved: refugees, helpers, general population etc.

This Research Topic presents an encouragement for further studies about this urgent topic through longitudinal studies, qualitative approaches, etc., given that one of the limitations of the present Research Topic is the use of cross-sectional designs.

It is likely that this war, like all previous ones, may have long-term negative mental health repercussions. We are confident that all the papers submitted in this Research Topic highlight the compounded and ripple effects of the Russian-Ukrainian war across the globe.

## Author contributions

MV drafted the editorial. AK, MT, CG, and OT revised and edited it. All authors contributed to the article and approved the version submitted.
